# Duration of symptoms before diagnosis in degenerative cervical myelopathy: A systematic review and meta-analysis

**DOI:** 10.1016/j.bas.2025.104252

**Published:** 2025-04-16

**Authors:** Ailish Malone, Maram Sofiany, Ghalia Dawood, James Wright, Rody Ryan, Caroline Treanor, Conor Gallagher, Warren Lenehan, Frank Doyle, Ciaran Bolger

**Affiliations:** a School of Physiotherapy, RCSI University of Medicine and Health Sciences, Ireland; b School of Medicine, RCSI University of Medicine and Health Sciences, Ireland; c National Neurosurgical Centre, Beaumont Hospital, Dublin, Ireland; d Department of Health Psychology, School of Population Health, RCSI, Ireland; e Department of Clinical Neuroscience, RCSI, Ireland

**Keywords:** Degenerative cervical myelopathy, Diagnosis, Diagnostic delay, Non-traumatic spinal cord injury

## Abstract

**Introduction:**

Degenerative cervical myelopathy (DCM), the commonest cause of spinal cord injury, can lead to progressive disability. Due to overlapping symptoms with other conditions and low awareness among healthcare professionals, many patients experience delayed diagnosis.

**Research question:**

What is the typical duration of symptoms of DCM before diagnosis?

**Materials and methods:**

We searched multiple databases for primary studies reporting duration of symptoms in people with confirmed diagnosis of DCM. Two independent reviewers screened titles, abstracts, full-text articles, extracted data and appraised study quality. We performed random-effects meta-analyses to pool duration of symptoms to presentation, diagnosis, and intervention.

**Results:**

We identified 78 studies from 18 countries, with 12,450 participants. Time from symptom onset to first clinical presentation (two studies, n = 232 participants) was 3.3 (95 % confidence interval, −0.3-6.8) months. Time from symptom onset to diagnosis (five studies, n = 897) was 15.0 (5.0–25.0) months. In 29 studies (n = 3052) that measured from symptom onset to surgery/pre-operative assessment the duration was 14.5 (12.1–17.0) months. Thirty-seven studies had an undefined endpoint, in which when pooled, duration of symptoms was 15.2 (12.4–18.0) months. Most studies did not define the symptoms at onset, however when onset was defined as first occurrence of myelopathic (upper motor neurone) symptoms (five studies, n = 1006), time to surgery was 10.7 (2.3–18.5) months.

**Discussion and conclusion:**

Most people with DCM experience symptoms for more than one year before diagnosis. These data may be useful to inform initiatives to promote early diagnosis. Standardised methodology would improve further research in this area.

## Highlights

• Diagnostic delay is commonly reported by people with DCM.
• This systematic review pooled data from 78 studies of 12,450 people in 18 countries.
• Time from first symptom onset to diagnosis or pre-operative assessment was 15 months.
• Time to surgery from onset of myelopathic symptoms was 11 months.
• There was variation in how studies reported and described duration of symptoms.

## 1 Background

Degenerative cervical myelopathy (DCM) is the most serious degenerative cervical spine pathology, causing potentially devastating and irreversible neurological disturbance, and the most common cause of spinal cord dysfunction in adults worldwide (Kalsi-Ryan et al., 2013). DCM encompasses a collection of pathological entities including spondylosis, degenerative disk disease, ossification of the posterior longitudinal ligament (OPLL), and ossification of the ligamentum flavum. Individually, or in combination, these processes cause compression of the cervical spinal cord, resulting in a clinical syndrome with typical features of gait imbalance, loss of hand dexterity and sphincter dysfunction (Tetreault et al., 2015a). The resulting disability can profoundly impact quality of life (Oh et al., 2017).

The detection and diagnosis of DCM is fraught with difficulty, with many patients reporting lengthy and convoluted pathways to diagnosis after first onset of symptoms (Behrbalk et al., 2013; Hilton et al., 2019). Once diagnosed, surgery offers significant but often incomplete gains in functional impairment, disability and pain in patients with moderate or severe DCM (Fehlings et al., 2017). A shorter duration of symptoms and less severe myelopathy preoperatively have been reported to be important predictors of achieving a good outcome (Tetreault et al., 2015b). Therefore, there is an urgency in detecting DCM.

The nature and typical duration of neurological symptoms that culminate in a diagnosis of DCM are poorly understood (Nouri et al., 2022). The accepted diagnostic criteria, of clinical (one symptom and one objective sign of upper motor neuron disturbance) and radiological (MRI evidence of spinal cord compression) evidence (Fehlings et al., 2013), require a threshold of neurological dysfunction. People with emerging DCM, and indeed their healthcare professionals, may not recognise the gravity of a slowly-progressive symptom burden, or attribute their symptoms to ageing or other mimics (Nouri et al., 2022). Service-level factors also contribute to diagnostic delay, with access to MRI and secondary care adding to the time from first presentation (Hilton et al., 2018).

A small number of studies have investigated time to diagnosis and diagnostic delay, employing study designs such as retrospective reviews of healthcare records (Behrbalk et al., 2013; Hilton et al., 2019) and online surveys (Pope et al., 2020). These studies identified a time to diagnosis of 18–26 months, albeit with significant variation and the potential confounder of incomplete recall. Many studies in DCM report duration of symptoms as a participant characteristic, alongside other demographic details and condition-specific evaluative measures. This provides a potentially wide pool of evidence from which to further investigate duration of symptoms, analyse the extent of diagnostic delay, and potentially contribute to knowledge about natural history, a top research priority in DCM (Nouri et al., 2022).

The aim of this systematic review was therefore to synthesise existing evidence to estimate pre-diagnosis duration of symptoms in people with DCM. A secondary objective was to describe and critically appraise the reporting of duration of symptoms in the literature, and consider these factors in the context of natural history.

## 2 Methods

### 2.1 Study design

This was a systematic review of primary studies. The protocol was prospectively registered with INPLASY on 22 June 2021 (registration 202160079), after initial search and before eligibility screening.

### 2.2 Eligibility criteria

Studies were eligible for inclusion if they were: 1) primary studies, 2) available in full text, 3) published or available in English, of 4) adults (minimum age 18 years) 5) with a confirmed diagnosis of DCM (or its synonyms) using objective clinical and radiological criteria (clinical symptom(s) and sign(s) of upper motor neurone lesion and radiological evidence of spinal cord compression) (Davies et al., 2018), and 6) quantitative report of duration of symptoms prior to diagnosis, assessment or intervention.

Studies were excluded if they reported spinal conditions other than DCM (radiculopathy, traumatic spinal injury, non-degenerative causes of spinal injury such as tumours or vascular lesions), or if they reported a mixed population of participants and it was not possible to extract data for those with DCM. Studies that reported secondary data analyses or additional follow-up on the same cohort of participants were also excluded. Where more than one report of the same dataset was published, the report with the largest number of participants was included in this review.

### 2.3 Search strategy

The following databases were searched: Medline (OVID), PubMed, EBSCO, Scopus, Embase, Web of Science, and CINAHL. No time restriction applied.

The search was performed on 31 March 2021 and repeated on 8 September 2023. An information specialist librarian contributed to the construction of the search. Search strategies are shown in Appendix 1.

### 2.4 Study selection

Three authors (MS, GD and AM) independently screened titles and abstracts for eligibility. Potentially eligible studies proceeded to full text review for final decision on inclusion. All full texts were reviewed by at least two authors (RR, JW, MS, GD, AM). Differences were resolved through discussion with a third author (AM or CT).

### 2.5 Quality appraisal

Methodological quality was assessed using the Crowe Critical Appraisal Tool, a 22-item general critical appraisal with proven construct validity and reliability (Crowe and Sheppard, 2011; Crowe et al., 2012). The CCAT was selected because it can be applied to a range of study designs, unlike other tools that are specific to study design, and our review did not restrict eligibility by study design.

Each paper was independently appraised by two authors (AM, MS, CG, CT and WL), with a maximum score out of 40 achievable. The two scoring authors then agreed a final score and overall quality, with banding as follows: Score 0–10, poor quality; 11–20, low quality; 21–30, fair quality; 31–40, good quality. Where authors differed significantly in their quality assessment, defined as a difference of more than 10 % or 5 points, a third author was consulted.

### 2.6 Data extraction

Two authors (JW and RR) independently extracted data for eligible studies, including study design; country where study took place; number, gender and age of participants; measure of severity of DCM; duration of symptoms (in months) and definition of duration of symptoms (start and end points). The two independently extracted data sheets were compared by a third author (AM) who collated the final data sheet for analysis.

### 2.7 Data analysis

Characteristics of included studies are presented in tabular format. The primary outcome, duration of symptoms, was explored for consistency of definition and reporting across the included studies, the review’s secondary objective. To do this, we extracted the text in each paper that described how they defined duration of symptoms, or if this was not available, we extracted information from elsewhere in the paper (for example, details in clinical assessment) that indicated how the start and end points of duration of symptoms were operationalised during data collection. We mapped the possible reporting options to key indicators in the natural history of DCM, namely, onset of first (any) symptom or specifically myelopathic (upper motor neuron) symptoms (Ozkan et al., 2022), and endpoint of time (first assessment, diagnosis or intervention). Based on these data, we created categories for how duration of symptoms was defined and reported in the included studies.

To meet the review’s primary aim, we took the following steps in data pooling and meta-analysis of duration of symptoms. Studies were pooled if their reporting of duration of symptoms was consistent, and they reported measures of central tendency (mean or median) and variance (interquartile range or standard deviation). Where a study did not report a standard deviation, this was imputed using Cochrane-recommended techniques (Higgins et al., 2022). As (mean) duration of symptoms was the primary outcome, we imputed missing data as follows. Where a paper did not report the mean but the median was available, we imputed the median as the mean. Where the standard deviation was not provided, we imputed the average standard deviation of the studies who did report it. Data were pooled using the Stata meta suite of commands. A random effects Hedges model was employed. Sensitivity analyses were undertaken to explore the impact of study quality (comparing poor- and low-quality studies defined as CCAT scores <21, with higher-quality studies) and time (comparing studies published before and after the year 2010, the decade before the first search was conducted for the review). Analysis was conducted in Stata IC 17 (StataCorp, Texas, USA).

## 3 Results

**Fig. 1:**
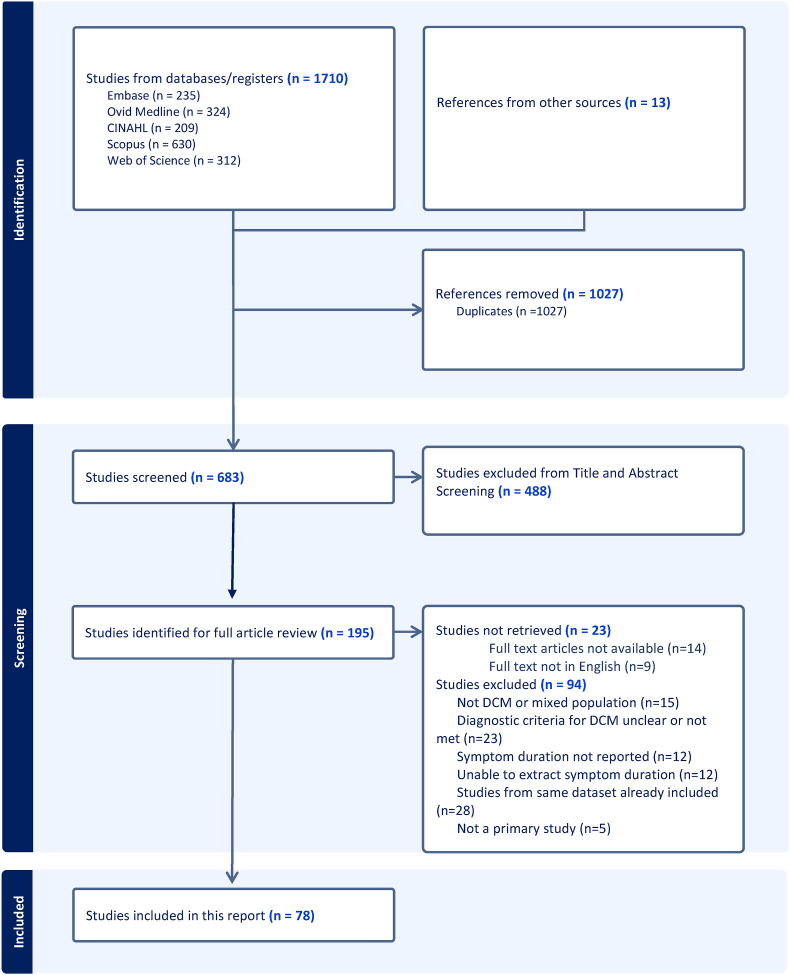
PRISMA 2020 flow diagram.

Fig. 1 shows the Preferred Reporting Items for Systematic Reviews and Meta-Analyses (PRISMA) flow diagram. After screening 683 titles and abstracts, 195 full texts were sought for assessment of eligibility. Fourteen papers were not available in full text and nine were not published in English, leaving 172 papers for consideration. Fifteen were excluded because the participants did not have DCM or the paper reported a mixed population and it was not possible to extract the DCM cohort. A further 23 papers were excluded for not clearly defining the diagnostic criteria for DCM, or not confirming evidence of both clinical and radiological features of DCM in their participants. Five papers were not primary studies. Twelve did not report duration of symptoms. A further 12 reported duration of symptoms, but it was not possible to extract their data quantitatively due to ambiguous reporting (for example, reporting by categories of less or more than three months, without useable aggregate data). A further 27 were secondary analyses from datasets already included. This left 78 papers for inclusion in the final review.

### 3.1 Characteristics of included studies

Regarding study design, 51 of 78 papers (65 %) (Behrbalk et al., 2013; Hilton et al., 2019; Ozkan et al., 2022), (Acharya et al., 2023; Aggarwal et al., 2016; Avadhani et al., 2010; Chen et al., 2019; Chen et al., 2020; Chen et al., 2016; Chen et al., 2015; Dalitz and Vitzthum, 2019; Edwards et al., 2002; Edwards et al., 2000; Fan et al., 2019; Fudo et al., 2023; Funaba et al., 2023; Gembruch et al., 2021; Heller et al., 2001; Jiang et al., 2022; Kato et al., 2008; Kawakami et al., 2002; Kihara et al., 2005; Kire et al., 2019; Lee et al., 2013; Lee et al., 2016; Li et al., 2019; Liu et al., 2011; Martin-Benlloch et al., 2003; Matsuda et al., 1999; Matsuyama et al., 2004; Morio et al., 2001; Naderi et al., 1998; Nagoshi et al., 2021; Notani et al., 2017; Pumberger et al., 2013; Sevki et al., 2004; Shin et al., 2023; Song et al., 2023; Tani et al., 2002; Uchida et al., 2014; Wada et al., 1995; Wada et al., 2001; Wei et al., 2019; Wei et al., 2018; Wen et al., 2012; Yamazaki et al., 2003) were retrospective studies, 22 (28 %) (Tetreault et al., 2015b; Archer et al., 2020; Bond et al., 2019; Evaniew et al., 2023; Furlan et al., 2011; Hirai et al., 2011; Hossam et al., 2013; Karpova et al., 2014; Liu et al., 2013; Lo, 2007; Loh et al., 2023; Machino et al., 2018; Marei et al., 2021; Matsukura et al., 2023; Paul et al., 2021; Pescatori et al., 2020; Suri et al., 2003; Suzuki et al., 2009; Tsitsopoulos et al., 2022; Zhang et al., 2011, 2016; Zika et al., 2020) were prospective studies, four (5 %) (Filimonova et al., 2023; Kalsi-Ryan et al., 2019; King et al., 2004; Malone et al., 2012) were cross-sectional, and one was ambispective (Martin et al., 2021) (Table 1). The majority (71 papers, 91 %) (Tetreault et al., 2015b; Ozkan et al., 2022), (Acharya et al., 2023; Aggarwal et al., 2016; Avadhani et al., 2010; Chen et al., 2019; Chen et al., 2020; Chen et al., 2016; Chen et al., 2015; Dalitz and Vitzthum, 2019; Edwards et al., 2002; Edwards et al., 2000; Fan et al., 2019; Fudo et al., 2023; Funaba et al., 2023; Gembruch et al., 2021; Heller et al., 2001; Jiang et al., 2022; Kato et al., 2008; Kawakami et al., 2002; Kihara et al., 2005; Kire et al., 2019; Lee et al., 2013; Lee et al., 2016; Li et al., 2019; Liu et al., 2011; Martin-Benlloch et al., 2003; Matsuda et al., 1999; Matsuyama et al., 2004; Morio et al., 2001; Naderi et al., 1998; Nagoshi et al., 2021; Notani et al., 2017; Pumberger et al., 2013; Sevki et al., 2004; Shin et al., 2023; Song et al., 2023; Tani et al., 2002; Uchida et al., 2014; Wada et al., 1995; Wada et al., 2001; Wei et al., 2019; Wei et al., 2018; Wen et al., 2012; Yamazaki et al., 2003), (Evaniew et al., 2023; Furlan et al., 2011; Hirai et al., 2011; Hossam et al., 2013; Karpova et al., 2014; Liu et al., 2013; Lo, 2007; Loh et al., 2023; Machino et al., 2018; Marei et al., 2021; Matsukura et al., 2023; Paul et al., 2021; Pescatori et al., 2020; Suri et al., 2003; Suzuki et al., 2009; Tsitsopoulos et al., 2022; Zhang et al., 2011, 2016; Zika et al., 2020; Filimonova et al., 2023), (Rajshekhar and Kumar, 2005) reported outcomes following decompressive surgery. Of these, one prospective study compared results of surgery to a non-surgical control (non-randomised) (Bond et al., 2019). The remaining 70 papers about surgery either reported single-arm results or compared two or more surgical techniques. Most studies came from Japan (19) (Edwards et al., 2000, 2002; Fudo et al., 2023; Funaba et al., 2023; Heller et al., 2001; Kato et al., 2008; Kawakami et al., 2002; Kihara et al., 2005; Matsuda et al., 1999; Matsuyama et al., 2004; Morio et al., 2001; Nagoshi et al., 2021; Notani et al., 2017; Pumberger et al., 2013; Tani et al., 2002; Uchida et al., 2014; Wada et al., 1995, 2001; Yamazaki et al., 2003; Archer et al., 2020; Hirai et al., 2011; Machino et al., 2018; Matsukura et al., 2023; Suzuki et al., 2009; King et al., 2004) and China (19) (Chen et al., 2015, 2016, 2019, 2020; Fan et al., 2019; Jiang et al., 2022; Li et al., 2019; Liu et al., 2011, 2013; Song et al., 2023; Wei et al., 2018, 2019; Wen et al., 2012; Yu et al., 2022; Zhang et al., 2011, 2016, 2018, 2022; Zhao et al., 2022), followed by India (seven), (Acharya et al., 2023; Aggarwal et al., 2016; Avadhani et al., 2010)^,^ (Kire et al., 2019)^,^ (Paul et al., 2021)^,^ (Suri et al., 2003)^,^ (Rajshekhar and Kumar, 2005), USA (six) (Edwards et al., 2000, 2002; Heller et al., 2001; Pumberger et al., 2013; Archer et al., 2020; King et al., 2004), Canada (six) (Bond et al., 2019; Evaniew et al., 2023; Furlan et al., 2011; Karpova et al., 2014; Kalsi-Ryan et al., 2019; Martin et al., 2021), three each from Germany (Ozkan et al., 2022; Dalitz and Vitzthum, 2019; Gembruch et al., 2021), and Korea (Lee et al., 2013, 2016; Shin et al., 2023), two from Turkey (Naderi et al., 1998; Sevki et al., 2004), Egypt (Hossam et al., 2013; Marei et al., 2021), and Singapore, (Lo, 2007; Loh et al., 2023); one multinational study (Tetreault et al., 2015b), and one each from Australia (Sekhon, 2003), Greece (Zika et al., 2020), Ireland (Malone et al., 2012), Israel (Behrbalk et al., 2013), Italy (Pescatori et al., 2020), Spain (Martin-Benlloch et al., 2003), Russia (Filimonova et al., 2023) and UK. (Hilton et al., 2019) The median sample size was 79 participants, ranging from 12 (Martin-Benlloch et al., 2003) to 2641 (Archer et al., 2020).

**Table 1:** Characteristics of included studies.

Study	Country	Design	Sample size	Age (years)				Duration of symptoms (months)		Definition of duration of symptoms	DCM severity outcome measure(s)	DCM severity score	Quality rating
				Mean	SD	Range		Mean	SD				
Acharya et al. (2023) (Acharya et al., 2023)	India	Retrospective case series	124	61.5	13.2	26	81	5.8	4.6	Undefined onset to surgery	mJOA	11.68 (SD, 2.12)	fair
Aggarwal et al. (2016) (Aggarwal et al., 2016)	India	Retrospective case series	78	nr				8.21		Undefined onset to surgery	mJOA	11.4	low
Archer et al. (2020) (Archer et al., 2020)	USA	Prospective	2641	60.4	11.4			12		Undefined	mJOA	12.4 (SD, 2.8)	fair
Avadhani et al. (2010) (Avadhani et al., 2010)	India	Retrospective case series	35	57.8		30	69	9.34		Undefined onset to surgery	Nurick	3.23	fair
Behrbalk et al. (2013) (Behrbalk et al., 2013)	Israel	Retrospective case series	42	52.5	12.6	20	77	26.4	27.6	Undefined onset to diagnosis	Nurick	2.9 (SD 0.53)	low
Bond et al. (2019) (Bond et al., 2019)	Canada	Prospective	122	54.8	12.6			24		Undefined onset to presentation	mJOA	16.0 (SD 0.97)	good
Chen et al. (2015) (Chen et al., 2015)	China	Retrospective case series	136	61.4		36	86	17.3		Undefined	JOA	Older = 7.25; Younger = 8.2	low
Chen et al. (2016) (Chen et al., 2016)	China	Retrospective case series	55	59.4	8.9			5.3	2.2	Undefined	JOA	10.7 (SD, 1.7)	fair
Chen et al. (2019) (Chen et al., 2019)	China	Retrospective case series	64	60.9	13.5			31.8	52.4	Undefined onset to surgery	JOA	6.2 (SD, 1.0)	fair
Chen et al. (2020) (Chen et al., 2020)	China	Retrospective case series	64	55.6	10.5	40	84	37.1	15.7	Undefined	mJOA	10.32 (SD 1.63)	low
Dalitz (2019) (Dalitz and Vitzthum, 2019)	Germany	Retrospective case series	43	53.9				20.7		Undefined	Nurick, JOA, EMS, Prolo, Cooper	JOA = 11.8	low
Edwards et al. (2000) (Edwards et al., 2000)	USA	Retrospective case series	18	54		34	79	48		Undefined	Nurick	2.7	low
Edwards et al. (2002) (Edwards et al., 2002)	USA	Retrospective case-control	26	53.5		35	72	9		Undefined onset to surgery	Nurick	2.1	low
Evaniew et al. (2023) (Evaniew et al., 2023)	Canada	Prospective	544	60.47	11.7			18.29	8.0	Undefined onset to diagnosis	mJOA	12.8 (SD 2.5)	good
Fan et al. (2019) (Fan et al., 2019)	China	Retrospective case series	89	58.97	5.8	37	78	14.64	8.0	Myelopathy symptoms to surgery	JOA	7.7 (SD 2.8)	fair
Filimonova et al. (2023) (Filimonova et al., 2023)	Russia	Cross-sectional	47	56.8	8.8	38	76	25.2	21.6	Undefined onset to surgery	n/r	n/r	fair
Fudo et al. (2023) (Fudo et al., 2023)	Japan	Retrospective	104	67.6				15.4∗		Undefined onset to surgery	n/r	n/r	low
Funaba et al. (2023) (Funaba et al., 2023)	Japan	Retrospective	103	72.7	9.8			19.7	29.8	Undefined onset to surgery	JOA	9.01 (SD 2.61)	fair
Furlan et al. (2011) (Furlan et al., 2011)	Canada	Prospective	81	57.0	1.4	32	88	25.2	2.7	Undefined onset to surgery	Nurick, mJOA	mJOA = 12.63	good
Gembruch et al. (2021) (Gembruch et al., 2021)	Germany	Retrospective case series	411	62.6	12.1	31	96	8.5		Undefined onset to surgery	mJOA	n/r	fair
Heller et al. (2001) (Heller et al., 2001)	USA	Retrospective case-control	26	55.5		34	79	18.5		Undefined onset to surgery	Nurick	2.25	low
Hilton et al. (2019) (Hilton et al., 2019)	UK	Retrospective case series	43	61.4	13.9			9.4	7.2	Undefined onset to diagnosis	Inferred mJOA	15.0	fair
Hirai et al. (2011) (Hirai et al., 2011)	Japan	Prospective	86	60.3	10.4	39	86	10.8	7.4	Undefined onset to surgery	JOA	9.8	low
Hossam et al. (2013) (Hossam et al., 2013)	Egypt	Prospective	30	48.9		35	65	12	10.1	Undefined	Benzel modification of JOA (BmJOA)	mJOA = 11.4	low
Jiang et al. (2022) (Jiang et al., 2022)	China	Retrospective	84	56.7	9.0			17.4	5.0	Undefined	JOA, Nurick	JOA = 11.7 (SD = 4.37) Nurick = 2.579 (SD = 0.936)	fair
Kalsi-Ryan et al. (2019) (Kalsi-Ryan et al., 2019)	Canada	Cross-sectional study	140	57.7	12.6			43.2	50.4	Undefined	mJOA	Stratified: Mild (15–17), n = 31; Mod (12–14), n = 57; Severe (<12), n = 59	fair
Karpova et al. (2014) (Karpova et al., 2014)	Canada	Prospective	134	55.6	1.0	29	82	25	2.8	Undefined	mJOA	Median mJOA = 13	fair
Kato et al. (2008) (Kato et al., 2008)	Japan	Retrospective case series	145	60.8	10.0	38	82	18.7	22.8	Undefined	JOA	Preserving C2 (N = 32) = 10.3 (2.2); Non-preserving C2 (N = 113) = 9.52 (2.8)	low
Kawakami et al. (2002) (Kawakami et al., 2002)	Japan	Retrospective case series	67	63	12.3	35	85	24.1	25.4	Undefined onset to surgery	JOA	JOA = 8.3 (SD, 3.3)	low
Kihara et al. (2005) (Kihara et al., 2005)	Japan	Retrospective case series	151	63		30	86	31		Undefined onset to diagnosis	JOA	8.1	low
King et al. (2004) (King et al., 2004)	USA	Cross-sectional study	79			29	85	66		Undefined	Nurick, mJOA	Nurick 3, mJOA 12	good
Kire et al. (2019) (Kire et al., 2019)	India	Retrospective case series	110	57	6.8	46	80	3.1	1.8	Undefined onset to presentation	mJOA, Nurick	mJOA (6.32, SD 0.87); Nurick (3.23 SD 0.71)	low
Lee, D (2013) (Lee et al., 2013)	Korea	Retrospective case-control	51	59.3	2.0	39	80	23.3	5.9	Undefined onset to surgery	JOA, Nurick	JOA = 12(SD, 0.6) Nurick = 1.7 (SD, 0.2)	low
Lee, SE (2016) (Lee et al., 2016)	Korea	Retrospective case series	70	53.9	9.2			31.7	42.2	Undefined onset to surgery	JOA	Group 1 JOA = 15.3; Group 2 = 11.7	low
Li, S (2019) (Li et al., 2019)	China	Retrospective case series	158	60.1	8.3			17.5	9.0	Undefined	JOA	11.6 (8–14, SD 1.29)	fair
Liu, T (2011) (Liu et al., 2011)	China	Retrospective case series	52	57.0	8.7			10.8	4.0	Undefined onset to surgery	JOA	JOA = 8.4 (SD 3.2)	low
Liu, F (2013) (Liu et al., 2013)	China	Prospective	94	53.1	10.8	36	77	13.7	12.8	Undefined onset to surgery	JOA	JOA = 9.0 (SD 2.6)	low
Loh et al. (2023) (Loh et al., 2023)	Singapore	Prospective	114	63	27.5				2.2	Undefined onset to surgery	JOA	10.6 (SD = 2.2)	fair
Machino et al. (2018) (Machino et al., 2018)	Japan	Prospective	1025	63.7	5.2	23	93	17.4	28.1	Undefined	JOA	JOA = 10.5 (SD 1.8(	fair
Malone et al. (2012) (Malone et al., 2012)	Ireland	Cross-sectional study	16	55.3	10.9	35	74	30		Undefined	mJOA, Nurick	11.2 (8–14, SD 1.8)	fair
Marei et al. (2021) (Marei et al., 2021)	Egypt	Prospective	40	51.8	7.7	36	74	11.3	10.9	Undefined onset to surgery	MJOA, Nurick	mJOA (11.225, SD = 1.687) Nurick (3.125, SD = 0.911)	low
Martin (2003) (Martin-Benlloch et al., 2003)	Spain	Retrospective case series	12	56		47	71	36		Undefined	Nurick	2.8	low
Martin et al. (2021) (Martin et al., 2021)	Canada	Ambispective	117	54.6	10.6			75.6	109.3	Undefined	mJOA	14.7 (SD = 2.1)	fair
Matsuda et al. (1999) (Matsuda et al., 1999)	Japan	Retrospective case series	41	61.4		20	81	13.4		Undefined onset to surgery	JOA	6.1 (older group), 6.5 (younger group)	low
Matsukara (2023) (Matsukura et al., 2023)	Japan	Prospective	395	63.7	11.4			43.4	65.1	Undefined onset to surgery	JOA	10.6 (SD = 2.9)	fair
Matsuyama et al. (2004) (Matsuyama et al., 2004)	Japan	Retrospective case series	44	61		38	76	26		Undefined	JOA	10.7	low
Morio et al. (2001) (Morio et al., 2001)	Japan	Retrospective case series	73	64.2	8.9	43	81	18.5	18.9	Undefined	JOA	10	low
Naderi et al. (1998) (Naderi et al., 1998)	Turkey	Retrospective case series	27	53.4		43	68	3.8		Undefined	mJOA	12 (range, 10–16)	low
Nagoshi et al. (2021) (Nagoshi et al., 2021)	Japan	Retrospective	80	64.5	11.7	32	86	41.8	54.5	Undefined	JOA	11.56 (SD = 2.79)	low
Notani et al. (2017) (Notani et al., 2017)	Japan	Retrospective	100	72.3	6.1	45	88	13.8	15.3	Undefined onset to surgery			
Ozkan et al. (2022) (Ozkan et al., 2022)	Germany	Retrospective	411	62.6	6.1	45	88		15.3	Myelopathy symptoms to surgery	mJOA	Median = 14.453	low
Paul et al. (2021) (Paul et al., 2021)	India	Prospective	34	56.6	12.1	31	96	17		Undefined onset to surgery	mJOA, Nurick	mJOA (11.294, SD = 2.558), Nurick (3.8, SD = 0.74)	fair
Pescatori et al. (2020) (Pescatori et al., 2020)	Italy	Prospective	60	57.5	7.8			25.33	17.4	Undefined	mJOA	N = 30: 13.2 N = 30: 7.3	fair
Pumberger et al. (2013) (Pumberger et al., 2013)	USA	Retrospective case series	248	59.0	10.6	19	72	15.1		Myelopathy symptoms to surgery	Nurick	Median = 1	low
Rajshekhar (2005) (Rajshekhar and Kumar, 2005)	India	Retrospective case series	72	49.7	13.3	16	86	21.4	18.1	Undefined	Nurick	4.24 (SD 0.43)	fair
Sevki et al. (2004) (Sevki et al., 2004)	Turkey	Retrospective case series	26	64.9		55	74	33.6		Undefined onset to surgery	Nurick, JOA	Nurick 3.5, JOA 7	low
Shin et al. (2023) (Shin et al., 2023)	Korea	Retrospective	59	65	11.7			7	11.0	Undefined onset to surgery	JOA, Nurick	JOA (9, SD = 3.4), Nurick (median = 2)	low
Song et al. (2023) (Song et al., 2023)	China	Retrospective	406	63.5	11.5	29	86	29	28.8	Undefined	JOA	10.55 (SD = 1.74)	fair
Suri et al. (2003) (Suri et al., 2003)	India	Prospective	146	47.1		17	76	11.7		Undefined	Nurick	Median = 3	low
Suzuki et al. (2009) (Suzuki et al., 2009)	Japan	Prospective	98	59.6	1.6			17.1	3.4	Undefined onset to surgery	JOA	11.1	fair
Tani et al. (2002) (Tani et al., 2002)	Japan	Retrospective case-control	26	64		43	77	37.5	53.7	Undefined onset to surgery	JOA	9.4	low
Tetreault (2015) (Tetreault et al., 2015b)	International	Prospective	757	56.5	11.9	21	87	26.8	39.3	Undefined	mJOA	12.52 (SD 2.74, 3–17 range)	fair
Tsitsopoulos et al. (2022) (Tsitsopoulos et al., 2022)	Sweden	Prospective	23	66.4	12.8	32	89	10∗		Undefined onset to surgery	n/r	n/r	fair
Uchida et al. (2014) (Uchida et al., 2014)	Japan	Retrospective case series	148	69.6	9.8	44	88	8.1	5.4	Myelopathy symptoms to surgery	JOA	11.0 (range, 3–15.5, SD 2.2)	fair
Wada et al. (1995) (Wada et al., 1995)	Japan	Retrospective case series	31	60	10.6			22.5	22.3	Undefined	JOA	10.1	low
Wada et al. (2001) (Wada et al., 2001)	Japan	Retrospective case-control	47	54.6	9.5			15.2	19.3	Undefined	JOA	7.7 (SD 2)	low
Wei et al. (2018) (Wei et al., 2018)	China	Retrospective case series	108	55.4	10.5	33	76	17.8	20.0	Undefined	JOA	10.2 (SD, 0.9)	fair
Wei et al. (2019) (Wei et al., 2019)	China	Retrospective case series	89	54.76	10.8	34	78	17.8	26.3	Undefined	JOA	10.1 (range, 8–12)	fair
Wen et al. (2012) (Wen et al., 2012)	China	Retrospective case series	229	56.4	11.4			4		Undefined onset to surgery	JOA	P = 10.4 (SD 3.0); P = 11.3 (SD 2.6); P = 10.2 (SD 2.7)	fair
Yamazaki et al. (2003) (Yamazaki et al., 2003)	Japan	Retrospective case series	64	64.6	12.0			25.6	30.6	Undefined	JOA	13.3 (SD 2.9)	low
Yew-Long (2007) (Lo, 2007)	Singapore	Prospective	46	57.6		36	84	11.4		Undefined	JOA	10.5	low
Yu et al. (2022) (Yu et al., 2022)	China	Retrospective	49	55.8	11.3			26.8	25.9	Undefined	mJOA	14.84 (SD = 1.78)	low
Zhang et al. (2011) (Zhang et al., 2011)	China	Prospective	52	56.3	6.4	45.0	67	16.1	6.8	Undefined	JOA	10.1	fair
Zhang et al. (2016) (Zhang et al., 2016)	China	Prospective	110	64.7	10.5	22	78	15.7	24.1	Undefined onset to surgery	JOA	9.9	fair
Zhang et al. (2018) (Zhang et al., 2018)	China	Retrospective case series	460	51.3	9.9	39	72	14.2		Myelopathy symptoms to surgery	JOA	10.4 (SD 2.2)	low
Zhang et al. (2022) (Zhang et al., 2022)	China	Retrospective	151	54.7	8.8	38	81	12∗	10.6	Undefined	mJOA	13.419 (SD = 2.056)	fair
Zhao et al. (2022) (Zhao et al., 2022)	China	Retrospective	33	53.5	11.9			43	19.0	Undefined	JOA	11.2 (SD = 1.6)	low
Zika et al. (2020) (Zika et al., 2020)	Greece	Prospective	36	50.8		39	70	21	8.3	undefined	mJOA	10.8 (SD 1.9)	fair

Abbreviations: EMS, European Myelopathy Score; JOA, Japanese Orthopaedic Association score; mJOA, modified Japanese Orthopaedic Association score; nr = not reported; SD, standard deviation. ∗median.

Three papers were published in the 1990s (Matsuda et al., 1999; Naderi et al., 1998; Wada et al., 1995), 18 between 2000 and 2009 (Edwards et al., 2000, 2002; Heller et al., 2001; Kato et al., 2008; Kawakami et al., 2002; Kihara et al., 2005; Martin-Benlloch et al., 2003; Matsuyama et al., 2004; Morio et al., 2001; Sevki et al., 2004; Tani et al., 2002; Wada et al., 2001; Yamazaki et al., 2003; Lo, 2007; Suri et al., 2003; Suzuki et al., 2009; King et al., 2004; Rajshekhar and Kumar, 2005; Sekhon, 2003), 33 between 2010 and 2019 (Behrbalk et al., 2013; Hilton et al., 2019; Tetreault et al., 2015b; Aggarwal et al., 2016; Avadhani et al., 2010; Chen et al., 2015, 2016, 2019; Dalitz and Vitzthum, 2019; Fan et al., 2019; Kire et al., 2019; Lee et al., 2013, 2016; Li et al., 2019; Liu et al., 2011, 2013; Notani et al., 2017; Pumberger et al., 2013; Uchida et al., 2014; Wei et al., 2018, 2019; Wen et al., 2012; Zhang et al., 2011, 2016, 2018; Bond et al., 2019; Furlan et al., 2011; Hirai et al., 2011; Hossam et al., 2013; Karpova et al., 2014; Machino et al., 2018; Kalsi-Ryan et al., 2019; Malone et al., 2012), and 24 between 2020 and 2023 (Ozkan et al., 2022; Acharya et al., 2023; Chen et al., 2020; Fudo et al., 2023; Funaba et al., 2023; Gembruch et al., 2021; Jiang et al., 2022; Nagoshi et al., 2021; Shin et al., 2023; Song et al., 2023; Yu et al., 2022; Zhang et al., 2022; Zhao et al., 2022; Archer et al., 2020; Evaniew et al., 2023; Loh et al., 2023; Marei et al., 2021; Matsukura et al., 2023; Paul et al., 2021; Pescatori et al., 2020; Tsitsopoulos et al., 2022; Zika et al., 2020; Filimonova et al., 2023; Martin et al., 2021).

The mean difference in study quality between the two independent raters was 0.6 points out of 40, or 1.5 %. Scores differed by more than 10 % (4 points) for 15 of 78 studies (19 %) and the final score for these papers was agreed by taking the mean score. Study quality was determined as follows. No papers were rated poor quality. Forty studies (51 %) were rated as low quality (CCAT score 11–20) (Behrbalk et al., 2013; Aggarwal et al., 2016; Chen et al., 2015, 2016; Dalitz and Vitzthum, 2019; Edwards et al., 2000, 2002; Fudo et al., 2023; Heller et al., 2001; Kato et al., 2008; Kawakami et al., 2002; Kihara et al., 2005; Kire et al., 2019; Lee et al., 2013, 2016; Liu et al., 2011; Martin-Benlloch et al., 2003; Matsuda et al., 1999; Matsuyama et al., 2004; Morio et al., 2001; Naderi et al., 1998; Nagoshi et al., 2021; Notani et al., 2017; Sevki et al., 2004; Shin et al., 2023; Tani et al., 2002; Wada et al., 1995, 2001; Yamazaki et al., 2003; Yu et al., 2022; Zhang et al., 2022; Zhao et al., 2022; Hirai et al., 2011; Hossam et al., 2013; Lo, 2007; Marei et al., 2021; Pescatori et al., 2020; Suri et al., 2003; Kalsi-Ryan et al., 2019; Rajshekhar and Kumar, 2005). Thirty-four studies (44 %) were rated fair quality (CCAT score 21–30) (Hilton et al., 2019; Tetreault et al., 2015b; Ozkan et al., 2022; Acharya et al., 2023; Avadhani et al., 2010; Chen et al., 2019, 2020; Fan et al., 2019; Funaba et al., 2023; Gembruch et al., 2021; Jiang et al., 2022; Li et al., 2019; Pumberger et al., 2013; Song et al., 2023; Uchida et al., 2014; Wei et al., 2018, 2019; Wen et al., 2012; Zhang et al., 2011, 2016, 2018; Archer et al., 2020; Karpova et al., 2014; Loh et al., 2023; Machino et al., 2018; Matsukura et al., 2023; Paul et al., 2021; Suzuki et al., 2009; Tsitsopoulos et al., 2022; Zika et al., 2020; Filimonova et al., 2023; Kalsi-Ryan et al., 2019; Malone et al., 2012; Martin et al., 2021). Four papers (5 %) were good quality (CCAT score 31–40) (Bond et al., 2019; Evaniew et al., 2023; Furlan et al., 2011; King et al., 2004). The most prevalent sources of bias within the studies were (1) selection bias or incomplete inclusion, particularly in retrospective studies which included only participants who had a particular surgical procedure without reference to the wider eligible cohort who had other surgeries or no surgery; (2) attrition bias, namely excluding large proportions of participants at the outset for incomplete follow-up; (3) unclear or inconsistent procedures for data collection, including non-standardisation of time-to-follow-up and lack of clarity on assessment procedures; (4) dichotomisation of outcome variables and/or predictor variables, without justifying cut-points; and (5) lack of sample size calculation or inadequate statistical power.

### 3.2 Participant characteristics

The 78 studies in this review included 12,450 participants. Sixty-nine studies reported the number of males and females in their samples; of these, 62.2 % (7091 of 11,401 participants) were male. The pooled mean age was 59.2 years (±2.3 years), with a mean minimum age of 34 years and a mean maximum age of 80 years. The youngest participant across all included studies was aged 16 years (Pumberger et al., 2013) and the oldest, 96 years (Gembruch et al., 2021).

Severity of DCM was measured using the original Japanese Orthopaedic Association (JOA) score (Yonenobu et al., 2001) in 41 studies (Chen et al., 2015, 2016, 2019; Dalitz and Vitzthum, 2019; Fan et al., 2019; Funaba et al., 2023; Jiang et al., 2022; Kato et al., 2008; Kawakami et al., 2002; Kihara et al., 2005; Lee et al., 2013, 2016; Li et al., 2019; Liu et al., 2011, 2013; Matsuda et al., 1999; Matsuyama et al., 2004; Morio et al., 2001; Naderi et al., 1998; Nagoshi et al., 2021; Notani et al., 2017; Sevki et al., 2004; Shin et al., 2023; Song et al., 2023; Tani et al., 2002; Uchida et al., 2014; Wada et al., 1995, 2001; Wei et al., 2018, 2019; Wen et al., 2012; Yamazaki et al., 2003; Zhang et al., 2011, 2016, 2018; Zhao et al., 2022; Hirai et al., 2011; Loh et al., 2023; Machino et al., 2018; Matsukura et al., 2023; Suzuki et al., 2009). Mean JOA scores ranged from 6.2 to 13.3, with a pooled mean of 9.9, corresponding to moderate disease severity using the classification of Yonenobu (Yonenobu et al., 2001). The modified JOA (mJOA) (Benzel et al., 1991) was reported in 22 studies (Hilton et al., 2019; Tetreault et al., 2015b; Ozkan et al., 2022; Acharya et al., 2023; Aggarwal et al., 2016; Chen et al., 2020; Kire et al., 2019; Yu et al., 2022; Zhang et al., 2022; Archer et al., 2020; Bond et al., 2019; Evaniew et al., 2023; Furlan et al., 2011; Hossam et al., 2013; Karpova et al., 2014; Marei et al., 2021; Paul et al., 2021; Pescatori et al., 2020; Zika et al., 2020; King et al., 2004; Malone et al., 2012; Martin et al., 2021). Mean mJOA scores ranged from 6.32 to 16, with a pooled mean of 12.4. This corresponds to moderate disease severity using the classification of Fehlings (Fehlings et al., 2013). The Nurick scale (Nurick, 1972) was reported in 21 studies (Behrbalk et al., 2013; Acharya et al., 2023; Avadhani et al., 2010; Dalitz and Vitzthum, 2019; Edwards et al., 2000, 2002; Heller et al., 2001; Jiang et al., 2022; Kire et al., 2019; Lee et al., 2013; Sevki et al., 2004; Shin et al., 2023; Furlan et al., 2011; Hossam et al., 2013; Marei et al., 2021; Paul et al., 2021; Suri et al., 2003; King et al., 2004; Malone et al., 2012; Martin et al., 2021; Rajshekhar and Kumar, 2005). Mean or median Nurick scores ranged from 1.7 to 4.2 with a pooled median grade of 3, corresponding to “extreme difficulty in walking that requires assistance and prevents full-time employment and occupation” (Nurick, 1972).

### 3.3 Duration of symptoms

There was considerable variation in how studies defined and reported duration of symptoms. Thirty-five studies (45 %) included a descriptor or definition of either the start or endpoint to which the duration referred. Five (6.4 %) (Ozkan et al., 2022; Fan et al., 2019; Pumberger et al., 2013; Uchida et al., 2014; Zhang et al., 2016), defined both the onset and endpoint; in these five studies, onset was defined as the first experience of specifically myelopathic (upper motor neurone) symptoms, and the endpoint was time of pre-operative assessment. The remaining 73 studies (93.6 %) did not describe the symptoms that defined the onset. Regarding the endpoint of duration of symptoms, 34 studies (37 %) defined it as time to surgery or pre-operative assessment. This included the aforementioned five studies who had defined the onset, and a further 29 (Acharya et al., 2023; Aggarwal et al., 2016; Avadhani et al., 2010)^,^ (Chen et al., 2020)^,^ (Edwards et al., 2002)^,^ (Fudo et al., 2023; Funaba et al., 2023; Gembruch et al., 2021; Heller et al., 2001)^,^ (Kawakami et al., 2002)^,^ (Lee et al., 2013)^,^ (Lee et al., 2016)^,^ (Liu et al., 2011)^,^ (Matsuda et al., 1999)^,^ (Notani et al., 2017)^,^ (Sevki et al., 2004)^,^ (Shin et al., 2023)^,^ (Tani et al., 2002)^,^ (Wen et al., 2012)^,^ (Zhang et al., 2018)^,^ (Furlan et al., 2011)^,^ (Hirai et al., 2011)^,^ (Liu et al., 2013)^,^ (Loh et al., 2023)^,^ (Marei et al., 2021)^,^ (Matsukura et al., 2023)^,^ (Suzuki et al., 2009)^,^ (Tsitsopoulos et al., 2022)^,^ (Filimonova et al., 2023) did not define the onset. Five studies (Behrbalk et al., 2013; Hilton et al., 2019; Kihara et al., 2005; Evaniew et al., 2023; Martin et al., 2021) reported time to diagnosis. Two studies (Kire et al., 2019; Bond et al., 2019) reported time to first clinical assessment after symptom onset. The remaining 37 studies (Tetreault et al., 2015b; Chen et al., 2016, 2019, 2020; Dalitz and Vitzthum, 2019; Edwards et al., 2000; Jiang et al., 2022; Kato et al., 2008; Li et al., 2019; Martin-Benlloch et al., 2003; Matsuyama et al., 2004; Morio et al., 2001; Naderi et al., 1998; Nagoshi et al., 2021; Song et al., 2023; Wada et al., 1995, 2001; Wei et al., 2018, 2019; Yamazaki et al., 2003; Yu et al., 2022; Zhao et al., 2022; Archer et al., 2020; Hossam et al., 2013; Karpova et al., 2014; Lo, 2007; Machino et al., 2018; Paul et al., 2021; Pescatori et al., 2020; Suri et al., 2003; Zhang et al., 2011; Zika et al., 2020; King et al., 2004; Malone et al., 2012; Rajshekhar and Kumar, 2005; Sekhon, 2003; Evaniew et al., 2020) did not define the endpoint to which their reporting of duration of symptoms referred. Two studies defined the endpoint as the time from onset (undefined) to study assessment (Kalsi-Ryan et al., 2019; Malone et al., 2012), but this was not described in relation to first presentation, diagnosis, or surgery. Most (35 studies) involved surgery, so we deduced that the endpoint was probably pre-operative assessment as this was the first timepoint for data collection.

To facilitate data synthesis of duration of symptoms, we created five categories from these descriptors:

1. Undefined symptom onset to first clinical assessment (two studies)
2. Undefined symptom onset to diagnosis (five studies)
3. Onset of defined DCM-specific symptoms to surgery or pre-operative assessment (five studies)
4. Undefined symptom onset to surgery/pre-operative assessment (29 studies)
5. Undefined symptom onset to undefined endpoint (37 studies)

Sixty-seven papers reported the mean duration of symptoms. The remaining eleven reported the median, or made it possible to calculate the median using raw data provided in the paper. Fifty-one papers reported a standard deviation for duration of symptoms in their data set.

Thirty-four studies reported the minimum (shortest) duration of symptoms in their dataset, ranging from 0.1 to 6 months, with a median of 1 month. The same 34 studies also reported the maximum (longest) duration of symptoms, which ranged from 24 to 912 months, with a median of 104 months.

#### 3.3.1 Meta-analysis of duration of symptoms

**Fig. 2:**
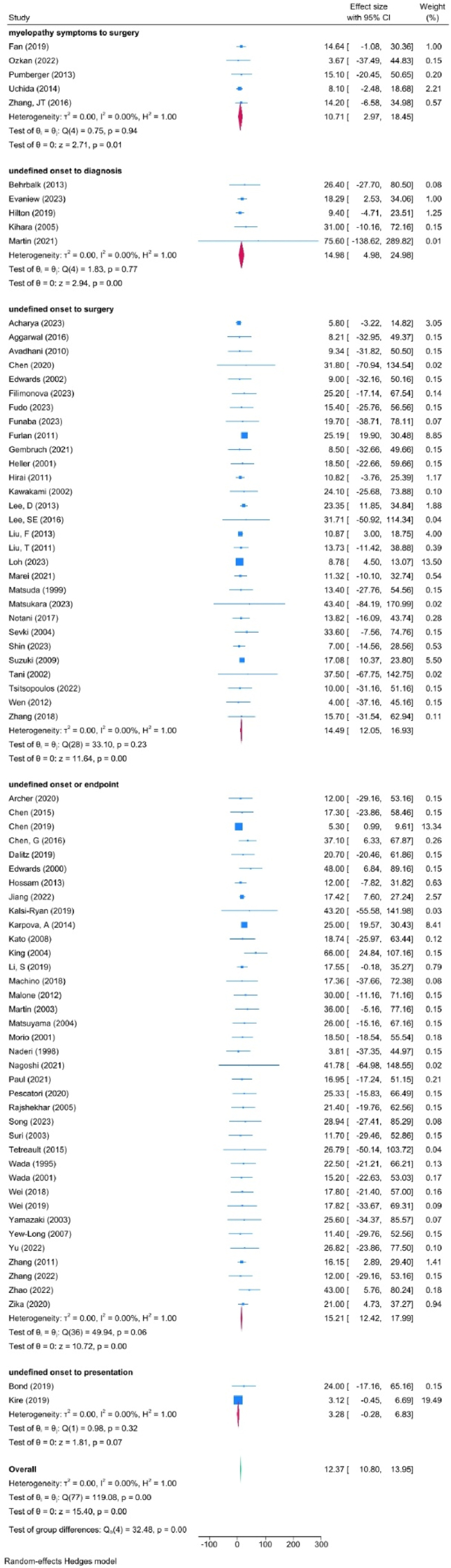
Forest plot of duration of symptoms in five categories of reporting.

Fig. 2 shows a forest plot of the findings of meta-analysis. The duration from onset of symptoms (undefined) to first clinical assessment was 3.3 months (95 % CI = −0.3 to 6.8 months; I^2^ = 0.00 %, n = 232, two studies). From onset (undefined) to diagnosis, the duration of symptoms was 15.0 months (95 % CI, 5.0–25.0 months; I^2^ = 0.00 %, n = 897, five studies). From onset (undefined) to surgery or pre-operative assessment, the duration of symptoms was 14.5 months (95 % CI, 12.1–17.0 months; I^2^ = 0.00 %, n = 3,052, 29 studies). When onset was defined as first occurrence of myelopathic symptoms, the time to surgery was 10.7 months (95 % CI, 3.2–18.5 months, I^2^ = 0.00 %, n = 1,006, five studies).

When undefined, but inferred as first symptom onset to pre-operative assessment, the duration of symptoms was 15.2 months (95 % CI, 12.4–18 months; I^2^ = 0.00 %, n = 7121; 37 papers).

We conducted two sensitivity analyses to explore for effect of year of publication (before and after 2010) and study quality (low-compared to fair- and good-quality studies). On the effect of publication year, the overall estimate of duration of symptoms was 19.3 months (95 % CI, 13.8–24.8 months) for studies published before 2010 and 14.4 months (95 % CI, 11.1–17.6 months) for studies published after 2010. On the effect of quality, the overall estimate of duration of symptoms was 14.5 months (95 % CI, 9.9–19.0 months) in low-quality studies and 15.4 months (95 % CI, 11.6–19.1 months) in fair- and good-quality studies.

## 4 Discussion

Early diagnosis of DCM is essential for guiding clinical management, achieving favourable outcomes and minimising disability. Detection of DCM is fraught with difficulty due to its non-specific signs and symptoms and poor general awareness of the condition. This systematic review contributes to knowledge about diagnostic delay by synthesising the available evidence on pre-diagnosis and pre-intervention duration of symptoms from a large body of evidence of 12,450 people with DCM in 18 countries worldwide. Consistent with clinical experience, we found evidence of prolonged duration of symptoms prior to diagnosis and surgery or pre-operative assessment, both from onset of first symptoms and, crucially, from occurrence of symptoms specific to myelopathy. The latter is particularly concerning as it represents potentially advanced disease. These findings underscore the importance of research to understand how DCM evolves vis-à-vis natural history, to raise awareness about DCM among healthcare professionals, and to develop interventions to support early detection of this debilitating condition.

There was significant ambiguity and variability in the reporting of duration of symptoms. Very few studies, representing 8 % of all participants, defined the onset with a description of symptoms specific to the upper motor neurone features that characterise DCM. Inconsistent or poorly defined reporting of duration of symptoms, particularly how they start, creates a gap in evidence for understanding natural history and creates difficulty in understanding how it might be detected sooner. Similarly, about half the studies, representing 58 % of all participants, did not clearly define an endpoint to which the duration of symptoms referred, though for most it was probably surgery. Of the studies that did define the endpoint, time to surgery (or pre-operative assessment) was reported for 33 % of participants. Time from symptom onset to diagnosis was reported for just 7 % of participants. This again limits our interpretation of diagnostic delay. Interestingly, our review found that the average time from (undefined) symptom onset to diagnosis was almost identical to the time to surgery or pre-operative assessment, namely 15 months. This might be explained by the predominance of studies in DCM involving surgery. Clinical guidelines recommend surgery for people with moderate to severe disease (Fehlings et al., 2017), so the similarity between time to diagnosis and time to pre-operative assessment or surgery may reflect the urgency of decompressive surgery. Additionally, more than two-thirds of the studies in our review were retrospective, involving data extraction from healthcare records. It is possible that the duration of symptoms at diagnosis was carried forward to pre-operative assessment.

Our review found indirect evidence that individuals who do not have surgery are under-represented in studies on DCM, as studies that focus on surgery necessarily restrict eligibility to those who opt for (or in retrospective studies, already had) it. Even though our eligibility criteria were open with regard to study aims and interventions, 91 % of studies had a primary aim to evaluate post-operative outcomes and 96 % of participants had surgery. This is unlikely to be consistent with typical clinical practice, particularly for people with mild DCM who may opt for surveillance or a trial of structured rehabilitation (Fehlings et al., 2017). The most recent clinical practice guideline recommends offering the option of surgery for people with mild DCM (Fehlings et al., 2017) with the goal to prevent further deterioration, but evidence does not support a prediction that deterioration is certain (Kadanka et al., 2017). Additionally, not everyone necessarily stabilises or improves after decompressive spinal surgery, which carries a 1-in-10 chance of worse functional scores after one year (Evaniew et al., 2023). This uncertainty makes it even more important to understand the natural history. Upon diagnosing DCM, it is reasonable that the focus should be on intervention and future outcomes, rather than on looking back at how symptoms evolved over a given timeframe. Nonetheless, this review also makes it clear that more studies need to define and report a comprehensive account of pre-diagnosis symptoms and their timeframes, similar to the work of Ozkan et al. (2022) (Ozkan et al., 2022) and Davies and colleagues (2020) (Davies et al., 2020).

Nonetheless, our findings suggest a timeline for evolution of DCM. The duration from symptom onset to when patients attended their first care assessment was 3.3 months, though this was reported by just three studies with a wide confidence interval and lacks precision as there were too few studies to estimate reliably for this outcome. From first onset to diagnosis, the average duration of symptoms was 15 months. Combining these findings suggests a delay of a year from first attending healthcare with an evolving DCM. The onset of DCM may be non-specific and not all patients initially present with myelopathic symptoms as listed in diagnostic criteria (Davies et al., 2018). A recent large retrospective analysis found that cervicobrachial neuralgia was the first symptom reported by 40 % of 411 patients with DCM (Ozkan et al., 2022), but this is non-specific to DCM and could reasonably be attributed to co-morbidities. Crucially, we found that when studies reported from the onset of specifically myelopathic symptoms, there was still a delay, with a duration (to pre-operative assessment) of 10.7 months. This indicates a timeframe when DCM could be detected clinically, but was instead possibly missed. This timeline comes from different cohorts and it is important to note the significant inter-individual variation. The longest duration of symptoms was over a decade in several studies (Tetreault et al., 2015b; Edwards et al., 2000; Kihara et al., 2005; Lee et al., 2016; Naderi et al., 1998; Nagoshi et al., 2021; Song et al., 2023; Wei et al., 2018, 2019; Wen et al., 2012; Furlan et al., 2011; Machino et al., 2018; Suri et al., 2003; Malone et al., 2012; Martin et al., 2021; Rajshekhar and Kumar, 2005). There is a need for a large prospective study to validate these timelines and explore the underlying ecological factors.

Duration of symptoms is generally accepted as an important predictor of outcome, with less favourable outcomes for those who have a longer pre-operative disease course. This in turn informs practice to recommend surgery without delay, especially for moderate or severe disease (Fehlings et al., 2017). Studies exploring symptom duration as a predictor of outcome after surgery have identified cut-points for better outcome ranging from four months (Tetreault et al., 2019) to twelve months (Archer et al., 2020) to two years (Levy et al., 2023) from symptom onset to surgery, whereas other studies have not found clear evidence of association between duration of symptoms and clinical outcomes (Evaniew et al., 2020; Asuzu et al., 2022) Such variation is unsurprising in the context of our review’s finding of variable and inconsistent definitions of symptom duration. A further problem in interpreting the effect of duration on outcome lies in how the outcome (functional improvement after surgery) is defined. The JOA or mJOA are commonly used but their minimum clinically important difference varies by disease severity and may over- or under-estimate the amount of recovery that is important to people living with DCM (Tetreault et al., 2015c). Furthermore, several studies that found an effect of symptom duration (Pumberger et al., 2013; Uchida et al., 2014; Wei et al., 2018; Suri et al., 2003; Zhang et al., 2016) define their outcome not on raw scores but by dichotomisation of the recovery rate, whereby the JOA or mJOA change score is computed as a percentage of baseline (Hirabayashi et al., 1981). This is problematic because the widely-used JOA recovery rate has never been validated against objective criteria or patient-reported outcomes. It was originally developed as a convenient means of expressing recovery for individual patients, but has since been widely adopted as a primary outcome. Interestingly, duration of symptoms does not correlate with disease severity (Pumberger et al., 2013; Kalsi-Ryan et al., 2019). The critical factor may be which specific symptoms and the rate of deterioration, rather than how long they have been present. The presence or absence of gait disturbance may be particularly important.Additionally, for stable disease, it is possible that patients may habituate to their symptoms over time (Kalsi-Ryan et al., 2019). Understanding these factors is a research priority in DCM (Nouri et al., 2022).

To address the gaps in reporting, we suggest clinicians and researchers include more comprehensive descriptors of the onset and evolution of symptoms. The recently-published DCM core outcome set describes symptoms according to indicators of neurological function and pain, and provides a useful framework for reporting first and subsequent symptoms (Davies et al., 2024a). To understand how symptoms evolve, defining the timelines from onset to (1) first presentation, (2) specialist assessment and investigation, and (3) diagnosis, while also monitoring disease severity with a validated outcome throughout these timelines, could contribute to knowledge on natural history. Additionally, there is a need for studies to address the gap in monitoring patients for whom there is uncertainty about surgery, or for whom it is not the preferred option.

Our systematic review has limitations. The unifying term for DCM was only recently defined in 2020 (Davies et al., 2024b). Before then, the condition was known by at least 11 different names. The heterogeneous and overlapping search terms, and inaccurate article indexing, creates imprecision in search strategies (Khan et al., 2020). Our initial broad search returned an unmanageable volume of records so we added an additional search term for “duration of symptoms” and its synonyms, but may have missed papers that reported it as a participant characteristic but not as a keyword or within the abstract. Additionally, although our review set out to explore time to diagnosis as the primary outcome, most studies did not report duration of symptoms to diagnosis, but rather to surgery. We pooled data based on how it was reported in the primary studies, but the variable reporting, particularly for retrospective studies of healthcare records, may have led to imprecision. Finally, some papers did not report the mean or standard deviation of duration of symptoms in their dataset and we had to impute it. We followed procedures for imputation recommended by the most recent Cochrane handbook (Higgins et al., 2022) but it must be acknowledged that imputation can be imprecise as it involves making assumptions about unknown data, particularly with regard to distribution, and this may affect the certainty of our findings.

## 5 Conclusion

Duration of symptoms from first onset to either diagnosis or pre-operative assessment is typically 15 months in DCM, and 11 months from onset of specifically myelopathic (upper motor neuron) symptoms. This corroborates patient experience of significant diagnostic delay. However, duration of symptoms is poorly defined in most studies. The imprecision may explain conflicting evidence about the extent to which symptom duration predicts outcome. Future studies should report duration of symptoms more precisely in terms of onset and endpoint, and describe the symptoms in more detail. This will inform natural history and contribute to understanding of the ecological factors underlying diagnostic delay. Finally, more evidence is needed on the disease course in people with DCM who do not have surgery.

## Declaration of competing interest

The authors declare that they have no known competing financial interests or personal relationships that could have appeared to influence the work reported in this paper.
