# Supplementary material for: Duration of symptoms before diagnosis in degenerative cervical myelopathy: A systematic review and meta-analysis
Source: Brain Spine. 2025 Apr 16;5:104252. doi: 10.1016/j.bas.2025.104252 (PMC12059671; doi:10.1016/j.bas.2025.104252)
Supplement: Multimedia component 1 [file mmc1.docx]

# Supplemental file 1. Search strategies.

## Ovid Medline

| No. | Query | Last Run Via | Results |
| --- | --- | --- | --- |
| 1 | cervical.tw. | Ovid Medline | 258,908 |
| 2 | Spinal Cord Compression/ | Ovid Medline | 11,886 |
| 3 | "cervical myelopathy".tw. | Ovid Medline | 3,139 |
| 4 | "cervical spondylotic myelopathy".tw. | Ovid Medline | 2,362 |
| 5 | "degenerative cervical myelopathy".tw. | Ovid Medline | 530 |
| 6 | 2 or 3 or 4 or 5 | Ovid Medline | 15,833 |
| 7 | "disease duration*".mp. | Ovid Medline | 25,589 |
| 8 | (disease adj3 duration*).mp. | Ovid Medline | 39,365 |
| 9 | "symptom* duration*".mp. | Ovid Medline | 4,804 |
| 10 | (symptom* adj3 duration*).mp. | Ovid Medline | 19,667 |
| 11 | "diagnostic delay*".mp. | Ovid Medline | 4,804 |
| 12 | (diagnostic adj3 delay*).mp. | Ovid Medline | 6,041 |
| 13 | 7 or 8 or 9 or 10 or 11 or 12 | Ovid Medline | 63,943 |
| 14 | 1 and 6 and 13 | Ovid Medline | 324 |

## Scopus

| No. | Query | Last Run Via | Results |
| --- | --- | --- | --- |
| 1 | TITLE-ABS ( cervical ) | Scopus | 311,294 |
| 2 | TITLE-ABS-KEY ( "spinal cord compression" ) | Scopus | 22,771 |
| 3 | TITLE-ABS ( "cervical myelopathy" ) | Scopus | 3,444 |
| 4 | TITLE-ABS ( "degenerative cervical myelopathy" ) | Scopus | 551 |
| 5 | TITLE-ABS ( "cervical spondylotic myelopathy" ) | Scopus | 2,671 |
| 6 | #2 OR #3 OR #4 OR #5 | Scopus | 26,759 |
| 7 | "disease duration*" | Scopus | 197,176 |
| 8 | disease W/3 duration* | Scopus | 225,816 |
| 9 | "symptom* duration*" | Scopus | 9,270 |
| 10 | symptom* W/3 duration* | Scopus | 41,879 |
| 11 | "diagnostic delay*" | Scopus | 17,403 |
| 12 | diagnostic W/3 delay* | Scopus | 23,190 |
| 13 | #7 OR #8 OR #9 OR #10 OR #11 OR #12 | Scopus | 280,408 |
| 14 | #1 & #6 & #13 | Scopus | 630 |

## Ebsco CINAHL

| No. | Query | Last Run Via | Results |
| --- | --- | --- | --- |
| 1 | TI cervical OR AB cervical | Ebsco Cinahl | 60,677 |
| 2 | (MH "Spinal Cord Compression") | Ebsco Cinahl | 2,430 |
| 3 | TI "cervical myelopathy" OR AB "cervical myelopathy" | Ebsco Cinahl | 1,000 |
| 4 | TI "degenerative cervical myelopathy" OR AB "degenerative cervical myelopathy" | Ebsco Cinahl | 194 |
| 5 | TI 'cervical spondylotic myelopathy' OR AB 'cervical spondylotic myelopathy' | Ebsco Cinahl | 866 |
| 6 | S2 OR S3 OR S4 OR S5 | Ebsco Cinahl | 3,867 |
| 7 | (MH "Disease Duration") | Ebsco Cinahl | 4,525 |
| 8 | TX "disease duration*" | Ebsco Cinahl | 12,288 |
| 9 | TX disease N3 duration | Ebsco Cinahl | 17,236 |
| 10 | TX "symptom* duration*" | Ebsco Cinahl | 2,704 |
| 11 | TX symptom* N3 duration* | Ebsco Cinahl | 11,236 |
| 12 | TX "diagnostic delay*" | Ebsco Cinahl | 2,097 |
| 13 | TX diagnostic N3 delay* | Ebsco Cinahl | 3,153 |
| 14 | TX "duration of symptom*" | Ebsco Cinahl | 5,043 |
| 15 | TX duration N3 symptom* | Ebsco Cinahl | 11,211 |
| 16 | S7 OR S8 OR S10 OR S11 OR S12 OR S13 OR S14 OR S15 | Ebsco Cinahl | 30,574 |
| 17 | S1 AND S6 AND S16 | Ebsco Cinahl | 111 |

## Web of Science

| No. | Query | Last Run Via | Results |
| --- | --- | --- | --- |
| 1 | cervical (Title) or cervical (Abstract) | Web of Science | 232,433 |
| 2 | "spinal cord compression" (All Fields) | Web of Science | 7,262 |
| 3 | "cervical myelopathy" (Title) OR "cervical myelopathy" (abstract) | Web of Science | 2,732 |
| 4 | "cervical spondylotic myelopathy" (Title) OR "cervical spondylotic myelopathy" (abstract) | Web of Science | 2,076 |
| 5 | "degenerative cervical myelopathy" (Title) OR "degenerative cervical myelopathy" (Abstract) | Web of Science | 539 |
| 6 | #2 OR #3 OR #4 OR #5 | Web of Science | 11,298 |
| 7 | #1 & #6 | Web of Science | 5,706 |
| 8 | "disease duration*" (All Fields) | Web of Science | 22,885 |
| 9 | "symptom* duration*" (All Fields) | Web of Science | 4,427 |
| 10 | "diagnositc delay*" (All Fields) | Web of Science | 4,825 |
| 11 | #8 OR #9 OR #10 | Web of Science | 32,956 |
| 12 | #1 & #6 & #11 | Web of Science | 97 |

## Embase

| No. | Query | Last Run Via | Results |
| --- | --- | --- | --- |
| 1 | cervical:ti,ab | Embase | 348,029 |
| 2 | 'spinal cord compression'/exp | Embase | 19,472 |
| 3 | 'cervical myelopathy':ti,ab | Embase | 4,056 |
| 4 | 'cervical spondylotic myelopathy':ti,ab | Embase | 3,064 |
| 5 | 'degenerative cervical myelopathy':ti,ab | Embase | 776 |
| 6 | #2 OR #3 OR #4 OR #5 | Embase | 24,941 |
| 7 | disease duration*' | Embase | 228,549 |
| 8 | (disease NEXT/3 duration*) | Embase | 233,001 |
| 9 | 'disease duration'/exp | Embase | 199,787 |
| 10 | symptom* duration*' | Embase | 8,519 |
| 11 | symptom* NEXT/3 duration* | Embase | 12,009 |
| 12 | diagnostic delay*' | Embase | 7,895 |
| 13 | (diagnostic NEXT/3 delay) | Embase | 6,973 |
| 14 | duration of symptom*' | Embase | 15,393 |
| 15 | (duration NEXT/3 symptom*) | Embase | 20,918 |
| 16 | 'delayed diagnosis'/exp | Embase | 18,075 |
| 17 | 'symptom duration'/exp | Embase | 18 |
| 18 | #7 OR #8 OR #9 OR #10 OR #11 OR #12 OR #13 OR #14 OR #15 OR #16 OR #17 | Embase | 279,835 |
| 19 | #1 AND #6 AND #18 | Embase | 640 |
| 20 | #19 NOT [medline]/lim | Embase | 235 |
